# Mps1-Ndc80: one interaction to rule them all

**DOI:** 10.18632/oncotarget.4837

**Published:** 2015-07-10

**Authors:** Jakob Nilsson

**Affiliations:** The Novo Nordisk Foundation Center for Protein Research, Faculty of Health and Medical Sciences, University of Copenhagen, Blegdamsvej, Copenhagen, Denmark

**Keywords:** Chromosome Section

Accurate chromosome segregation during mitosis is ensured by a sophisticated surveillance mechanism, the spindle assembly checkpoint (SAC) that only allows sister chromatid separation once all kinetochores have bound to microtubules. A major unresolved question has been how the SAC senses microtubule binding to kinetochores and how this couples with turning the checkpoint off. Two papers now show that the kinetochore binding site for microtubules and the checkpoint kinase Mps1 overlap providing an elegant answer to this question.

**Figure 1:**
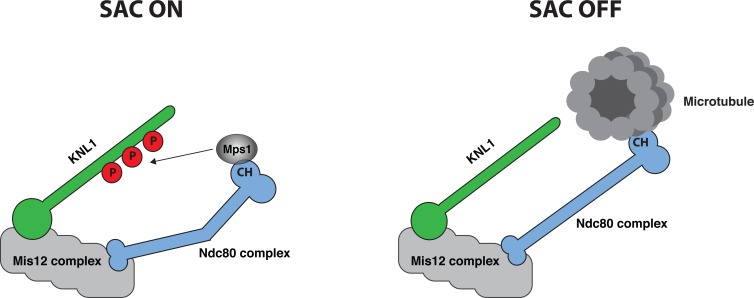
Regulation of Mps1 localization by microtubule binding. Mps1 localizes to unattached kinetochores through a direct interaction between Mps1 and the Ndc80 CH domain. This brings Mps1 in proximity of KNL1, which promotes phosphorylation of MELT repeats. Upon microtubule binding Mps1 is displaced and the Ndc80 CH moves away from KNL1 resulting in SAC silencing.

Kinetochores are large protein assemblies at the constriction point of chromatids that perform two key functions during mitosis. First, kinetochores can bind directly to microtubules of the mitotic spindle, a requirement for sister chromatid separation. Second, unattached kinetochores activate the SAC by recruiting checkpoint proteins and their localization to this structure results in the generation of a diffusible “wait anaphase” signal. The KMN network is an outer kinetochore complex composed of KNL1, the Mis12 complex and the Ndc80 complex that executes and integrates these two important functions of kinetochore biology (Figure 1). The KMN network can bind directly to microtubules with the major microtubule binding activity residing in the Ndc80 protein. In particular, the calponin homology (CH) domain and an unstructured tail of the Ndc80 protein binds to microtubules with some additional contribution from the Nuf2 protein that is a stable binding partner of Ndc80.

The KMN network is also required for SAC signaling because it contains the binding sites for checkpoint proteins at the kinetochore. Checkpoint proteins are recruited to kinetochores in a hierarchical manner with the Mps1 checkpoint kinase required for the localization of all downstream components. An initiating checkpoint event is Mps1 phosphorylation of multiple methionine-glutamate-leucine-threonine (MELT) repeats in KNL1 that creates binding sites for the Bub1-Bub3 checkpoint complex [1]. Bub1-Bub3 then promotes the localization of the BubR1-Bub3 and Mad1-Mad2 checkpoint complexes that by a poorly understood mechanism generates the “wait anaphase” signal.

Although it has been clear that end-on microtubule binding to kinetochores results in removal of checkpoint proteins and SAC silencing, the mechanistic details of how this is achieved has been unknown. Building on previous work showing an interaction between Mps1 and the Ndc80 complex in yeast [2] and human cells [3], the Kops and Yu labs now provide important detailed insight into this interaction and its regulation [4, 5]. The first 200 amino acids of Mps1 specify its kinetochore localization and harbor a TPR domain and an N-terminal extension (NTE). The groups show that this part of Mps1 directly binds to the Ndc80 complex and interacts strongly with the Ndc80 CH domain. Importantly, binding of microtubules to Ndc80 *in vitro* and in cells prevents Mps1 binding showing direct competition (Figure 1). In agreement with this, mutating residues in the Ndc80 CH domain that are required for microtubule binding or residues close to the microtubule binding site also reduces Mps1 binding and localization. Not only is the interaction between Mps1 and the Ndc80 complex regulated by microtubule binding but also through phosphorylation of both Mps1 and the Ndc80 complex. The Kops lab shows that phosphorylation of the NTE by Mps1 strongly increases the affinity for Ndc80 CH while the Yu lab identifies a short region C-terminal to the TPR domain that interacts with Nuf2 in a phospho-dependent manner. Furthermore, phosphorylation by Aurora B of the N-terminal tail of Ndc80 increases the affinity for Mps1 while preventing microtubule binding. The exact details of how the different phosphorylations are regulated and how they modulate the Mps1-Ndc80 interaction are important future goals.

Is direct competition between microtubules and Mps1 for the Ndc80 complex the only physical mechanism in place to turn the checkpoint off? Work in budding yeast from the Joglekar lab now shows that the separation of Mps1 from Spc105 (KNL1 in yeast) brought about by changes in kinetochore architecture due to microtubule binding contributes to checkpoint silencing [6]. By carefully anchoring Mps1 at defined positions within the kinetochore, they show that Mps1 needs to be in proximity of Spc105 to phosphorylate it and activate the checkpoint. Interestingly, artificially maintaining Mps1 close to the Ndc80 CH domain did not prevent SAC silencing once kinetochores bound microtubules because the Ndc80 CH domain becomes physically separated away from Spc105. This mechanism is unlikely to be specific for yeast because previous work have shown that the Ndc80 CH domain moves away from KNL1 upon microtubule binding and that intra-kinetochore stretch correlates with SAC activity [7]. The exact localization of the KNL1 MELT repeats with respect to Mps1 in human cells and how microtubule-binding controls this will be interesting to determine.

We clearly now have a much better understanding of how microtubule binding to kinetochores turns off SAC signaling by hampering Mps1 phosphorylation of KNL1. Whether microtubule binding affects other aspects of SAC signaling remains to be explored.
